# Supplementary material for: Wnt and Src signals converge on YAP‐TEAD to drive intestinal regeneration
Source: EMBO J. 2021 May 5;40(13):e105770. doi: 10.15252/embj.2020105770 (PMC8246259; doi:10.15252/embj.2020105770)
Supplement: Supplementary file 1 — Appendix [file EMBJ-40-e105770-s002.pdf]

Appendix – Guillermin *et al* 2021: Wnt and Src signals converge on YAP-TEAD to drive intestinal regeneration

Table of Contents

Appendix Figure S1. Gradients of *Yap* mRNA and YAP protein expression indicate transcriptional control of *Yap* by Wnt.

Appendix Figure S2. Wnt regulation of *Yap* and *TEAD1/2/4* expression in organoids and intestinal crypts *in vivo*.

Appendix Figure S3. *Lats1/2* double knockout organoids maintain expression of Sox9 and continue to undergo cell proliferation after 5 days of culture.

Appendix Figure S4. Expression of nlsYAP^{5SA} in *Apc-/- p53-/-* cKO organoids does not impair growth despite multiple passages in culture.

Appendix Figure S5. Expression of nlsYAP^{5SA} in *Apc-/- p53-/-* cKO organoids does not impair growth after subcutaneous implantation into nude mice.

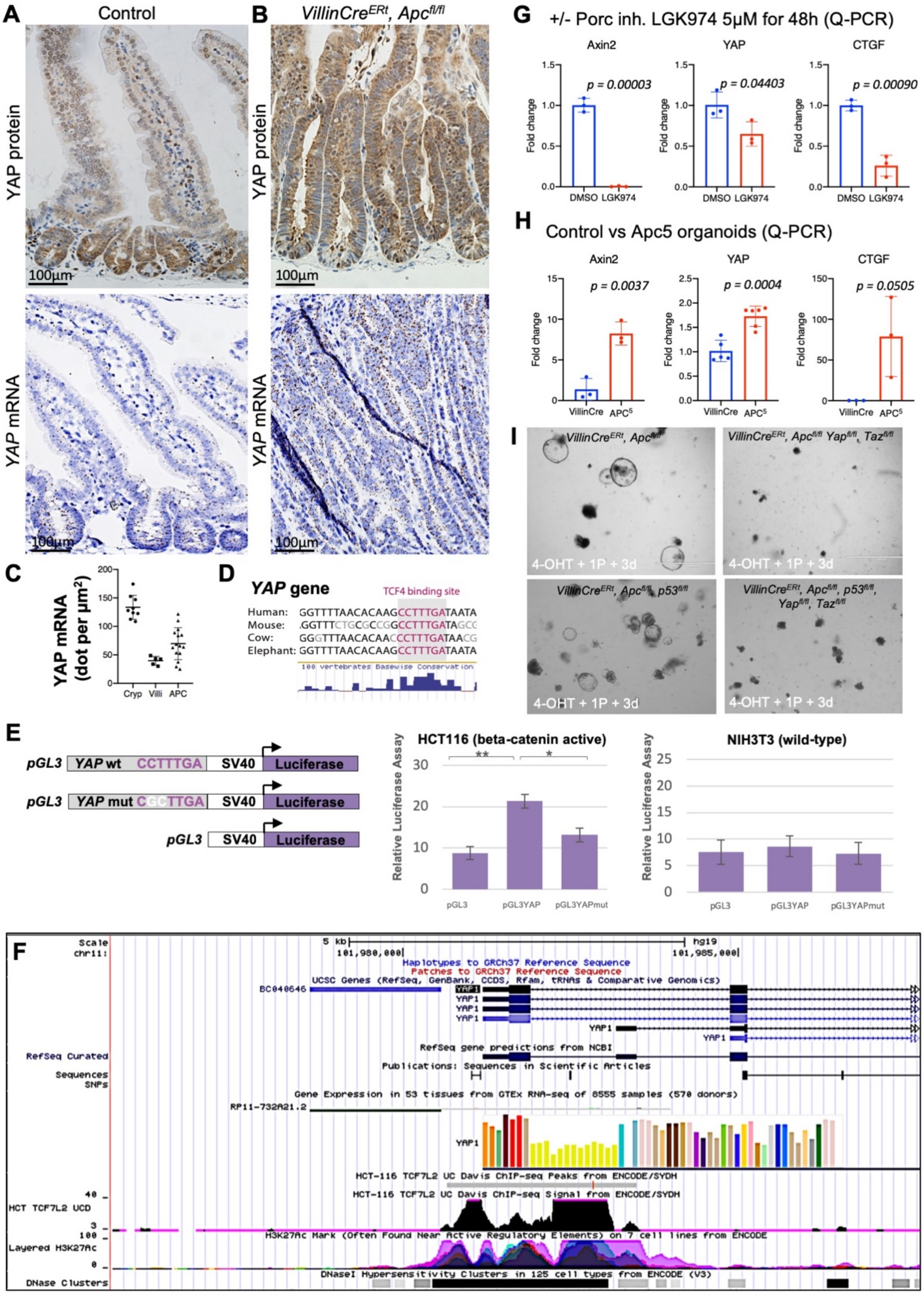

Appendix Figure S1. Gradients of *Yap* mRNA and YAP protein expression indicate transcriptional control of *Yap* by Wnt.

A) Wild-type mouse small intestine immunostained for YAP protein reveals strong staining in the intestinal crypt that declines along the villus, with YAP localised primarily to the cytoplasm. RNAscope *in situ* hybridisation for *Yap* mRNA reveals an identical gradient of expression along the crypt-villus axis.

B) Apc^{Min} intestine immunostained for YAP protein reveals strong expression throughout the tumour, with most YAP localised to the cytoplasm (note blue nuclei). RNAscope *in situ* hybridisation for *Yap* mRNA reveals an identical pattern of uniform high expression throughout the tumour. n > 10 tumours analysed (see also Fig S1).

C) Quantification of *Yap* mRNA expression level in the wildtype crypt versus the villus reveals a 3-fold average difference, while Apc^{Min} intestinal tumours show intermediate levels of *Yap* mRNA. n = 6 independent experiments. A paired 2-tailed t-test reveals a statistically significant difference ($p < 0.005$) between crypt and villi.

D) A conserved TCF4 binding site in the *Yap* promoter region explains the regulation of *Yap* transcription by Wnt signalling.

E) Schematic diagram of a luciferase reporter gene assay in which the human *YAP1* promoter drives expression of the firefly luciferase gene. Mutation of the conserved TCF4 binding site is shown. A single TCF4 site in *YAP1* is necessary for Wnt-dependent gene transcription in luciferase reporter assays in colorectal cancer cells.

F) Schematic diagram of the *YAP1* promoter showing TCF4 Chromatin IP data curated by the ENCODE consortium.

G) Treatment of wild-type murine intestinal organoids with the Wnt secretion inhibitor (Porc inhibitor LGK974) at 5μM for 48h causes inhibition of Wnt target gene expression (*Axin2* mRNA) as well as reduction in the expression of *Yap* and *CTGF* mRNAs as determined by quantitative RT-PCR assays. Statistical analysis was performed with a paired 2-tailed t-test.

H) *Apc5* mutant murine intestinal organoids show increased expression of a Wnt target gene (*Axin2* mRNA) along with increased expression of *Yap* and *CTGF* compared to wild-type control organoids. Statistical analysis was performed with a paired 2-tailed t-test.

I) Either $VillinCre^{ERt2}$, $Apc^{fl/fl}$ or $VillinCre^{ERt2}$, $Apc^{fl/fl}$, $p53^{fl/fl}$ tumour derived organoids grow to form spheres in Matrigel after deletion with tamoxifen (4-OHT), one passage and 3 days of culture. Knockout of YAP/TAZ in either $VillinCre^{ERt2}$, $Apc^{fl/fl}$ or $VillinCre^{ERt2}$, $Apc^{fl/fl}$, $p53^{fl/fl}$, $Yap^{fl/fl}$, $Taz^{fl/fl}$ organoids prevents growth in Matrigel after tamoxifen (4-OHT), one passage and 3 days of culture.

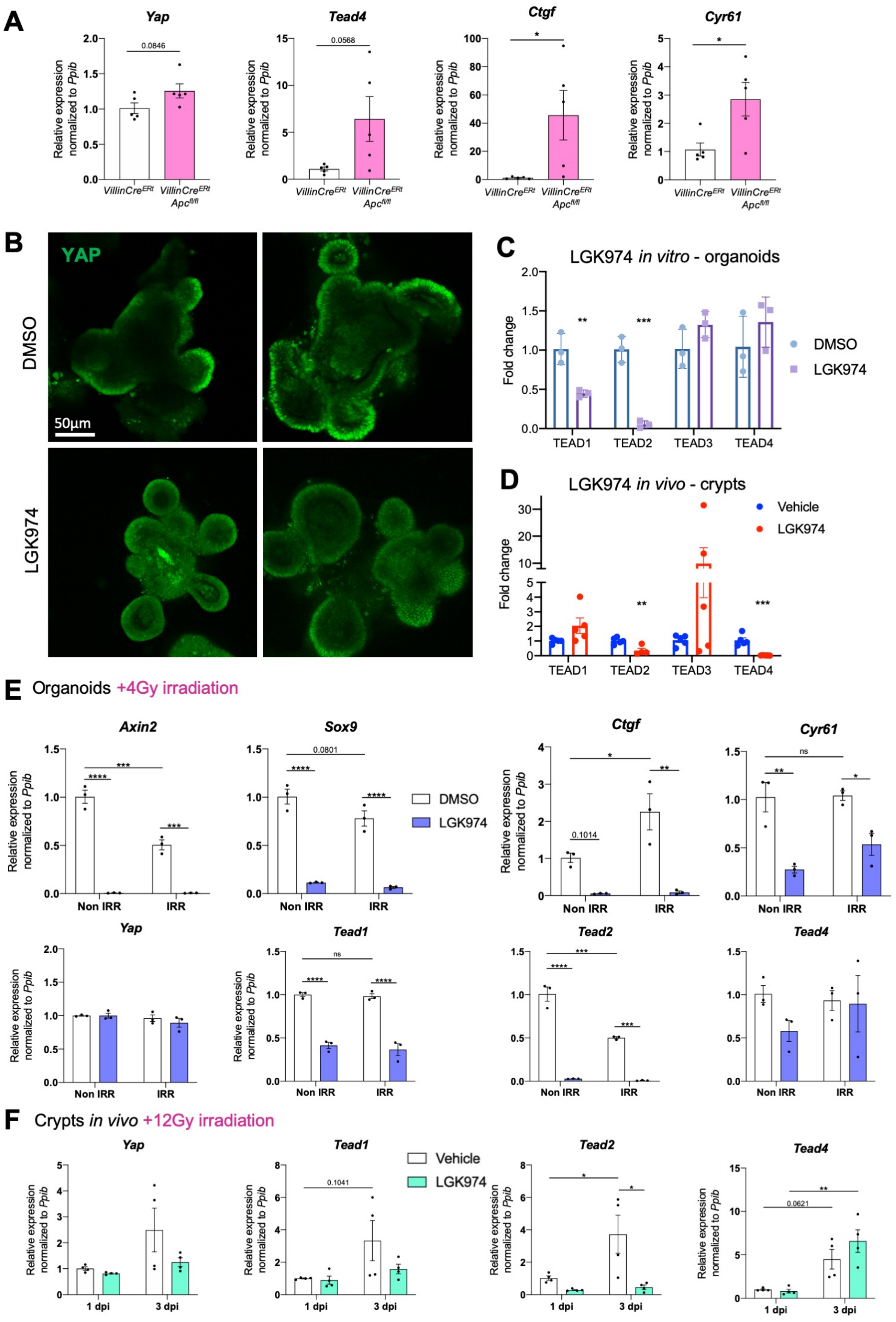

Appendix Fig S2. Wnt regulation of *Yap* and *TEAD1/2/4* expression in organoids and intestinal crypts *in vivo*.
A) Q-PCR analysis of *Yap, TEAD4, Ctgf* and *Cyr61* mRNA expression in control and *Apc5* mutant organoids cultured in Matrigel for 3 days. Note that the mild induction of *Yap* and stronger induction of *TEAD4*, which together may account for the increase in *Ctgf* and *Cyr61.*
B) Murine intestinal organoids immunostained for YAP after treatment with DMSO (control) or Porcupine inhibitor LGK974. Note that the gradient of YAP expression in crypt buds is abolished upon LGK974 treatment.
C) Q-PCR analysis of DMSO and LGK974 treated organoids reveals a strong downregulation of *TEAD1* and *TEAD2* expression, but not *TEAD3* or *TEAD4*.
D) Q-PCR analysis of intestinal crypts isolated from DMSO and LGK974 treated animals reveals a strong downregulation of *TEAD1*, *TEAD2,* and *TEAD4* expression, but not *TEAD3*.
E) Q-PCR analysis of organoids treated with 4Gy irradiation in culture with either DMSO or Porcupine inhibitor LGK974.
F) Q-PCR analysis of intestinal crypts isolated from animals treated with 12Gy irradiation with either Vehicle or Porcupine inhibitor LGK974.

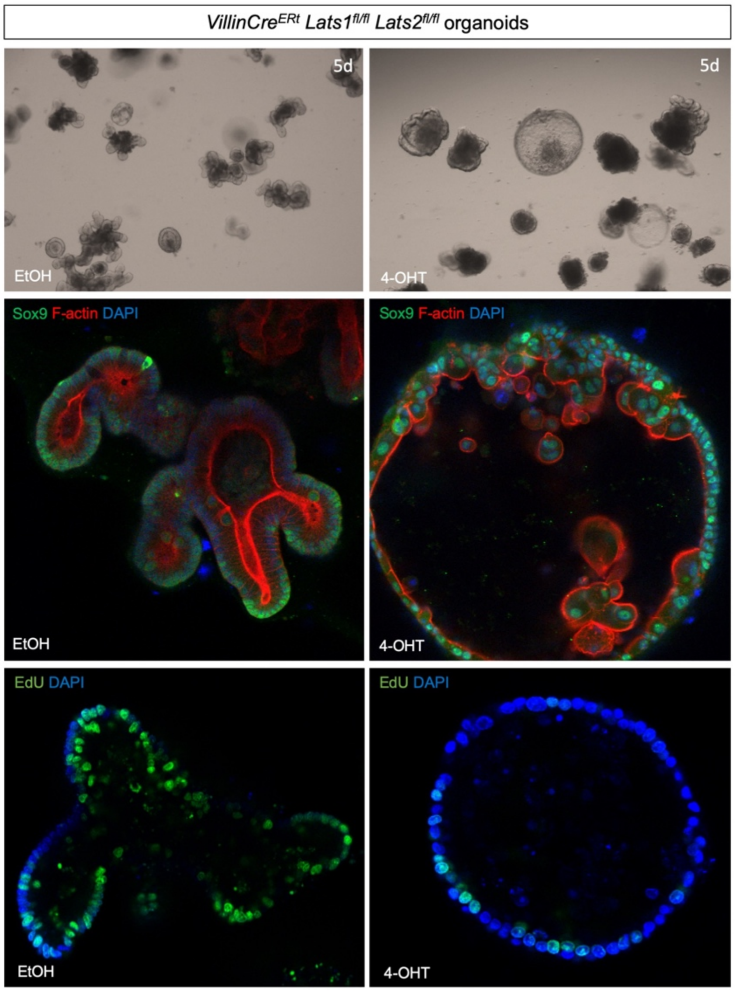

Appendix Fig S3. *Lats1/2* double knockout organoids maintain expression of Sox9 and continue to undergo cell proliferation after 5 days of culture.
Murine intestinal organoids cultured from *VillinCre*^{ERt} *Lats1*$^{fl/fl}$ *Lats2*$^{fl/fl}$ double knockout *(Lats1/2 dKO)* animals treated with either ethanol (EtOH) or 4-hydroxytamoxifen (4-OHT) to induce homozygous deletion. After 5 days in culture, the Lats1/2 dKO organoids grew to form large spheres that immunostained positively for Sox9 and continued to incorporate EdU, indicating ongoing cell proliferation.

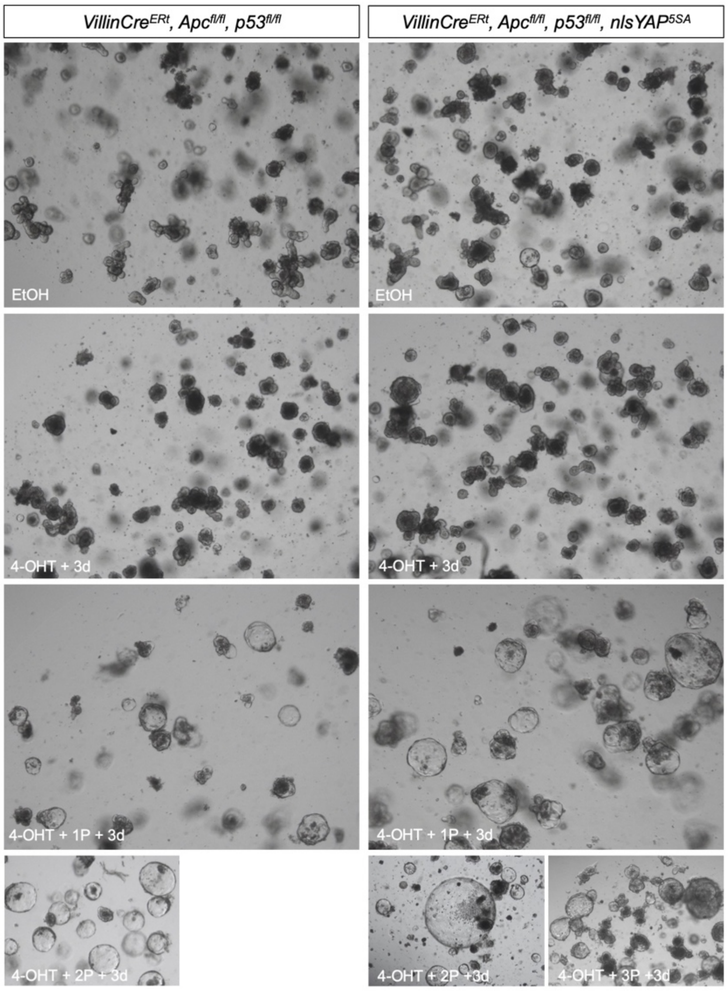

Appendix Fig S4. Expression of nlsYAP^{5SA} in *Apc-/- p53-/-* cKO organoids does not impair growth despite multiple passages in culture.
Murine intestinal organoids cultured from *VillinCre*^{ERt} *Apc*$^{fl/fl}$ *p53*$^{fl/fl}$ animals with or without co-expression of *Rosa26>loxSTOPlox>nlsYAP*^{5SA} reveals that nlsYAP5SA expression does not impair organoid growth after treatment with 4-OHT, even after multiple passages and days in culture.

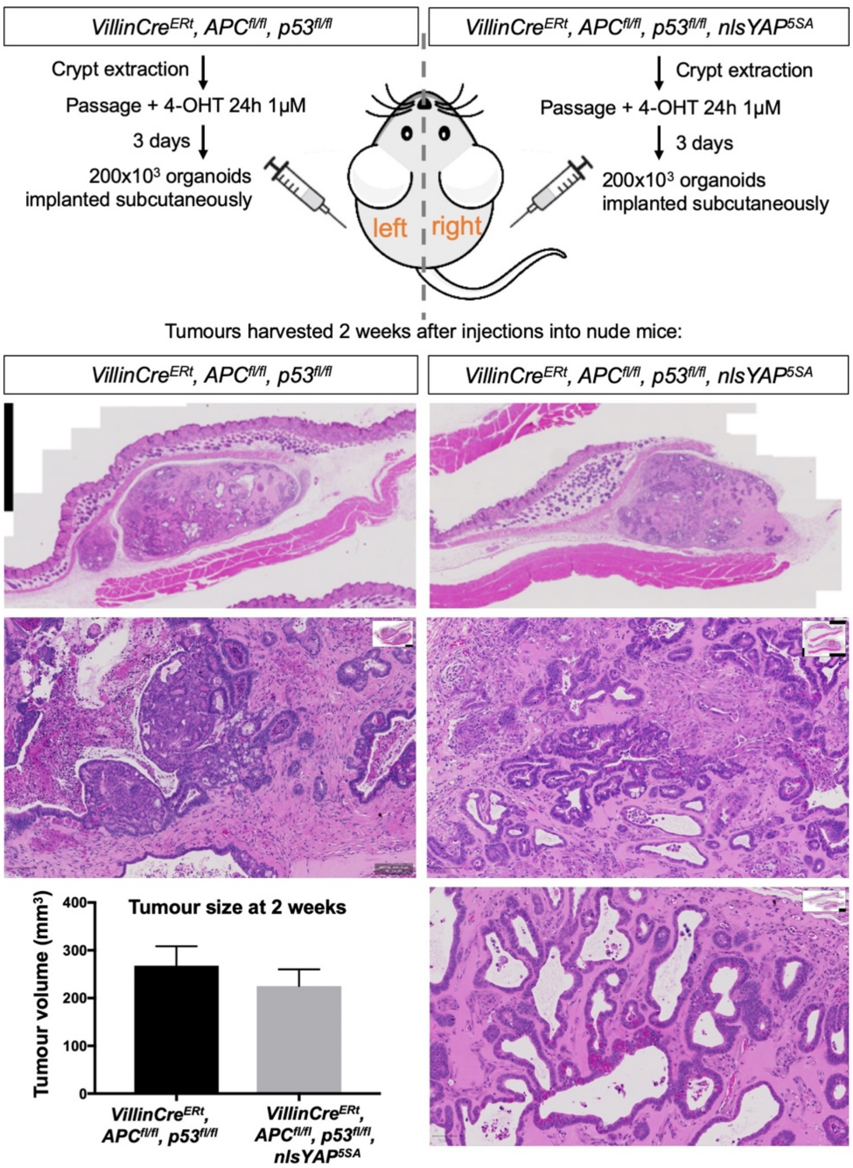

Appendix Fig S5. Expression of nlsYAP^{5SA} in *Apc-/- p53-/-* cKO organoids does not impair growth after subcutaneous implantation into nude mice.
Murine intestinal organoids cultured from *VillinCre^{ERt} Apc$^{fl/fl}$ p53$^{fl/fl}$* animals with or without co-expression of *Rosa26>loxSTOPlox>nlsYAP^{5SA}* were treated with 4-OHT and implanted subcutaneously into nude mice. Expression of *nlsYAP^{5SA}* did not impair tumour formation *in vivo* after 2 weeks.
