# Supplementary material for: Wnt and Src signals converge on YAP‐TEAD to drive intestinal regeneration
Source: EMBO J. 2021 May 5;40(13):e105770. doi: 10.15252/embj.2020105770 (PMC8246259; doi:10.15252/embj.2020105770)
Supplement: Supplementary file 2 — Expanded View Figures PDF [file EMBJ-40-e105770-s001.pdf]

Expanded View Figures

Figure EV1. YAP, TAZ and CTGF expression patterns in the human intestine, adenomas and invasive CRC.

A Normal human small intestines stained for YAP, TAZ and CTGF.
B Normal human colons stained for YAP, TAZ and CTGF.
C Human colorectal adenomas stained for YAP, TAZ and CTGF.
D Human invasive colorectal carcinomas stained for YAP, TAZ and CTGF.
E Magnified view of (A) showing nuclear YAP and CTGF expression in crypt base stem cells.

 © 2021 The Authors

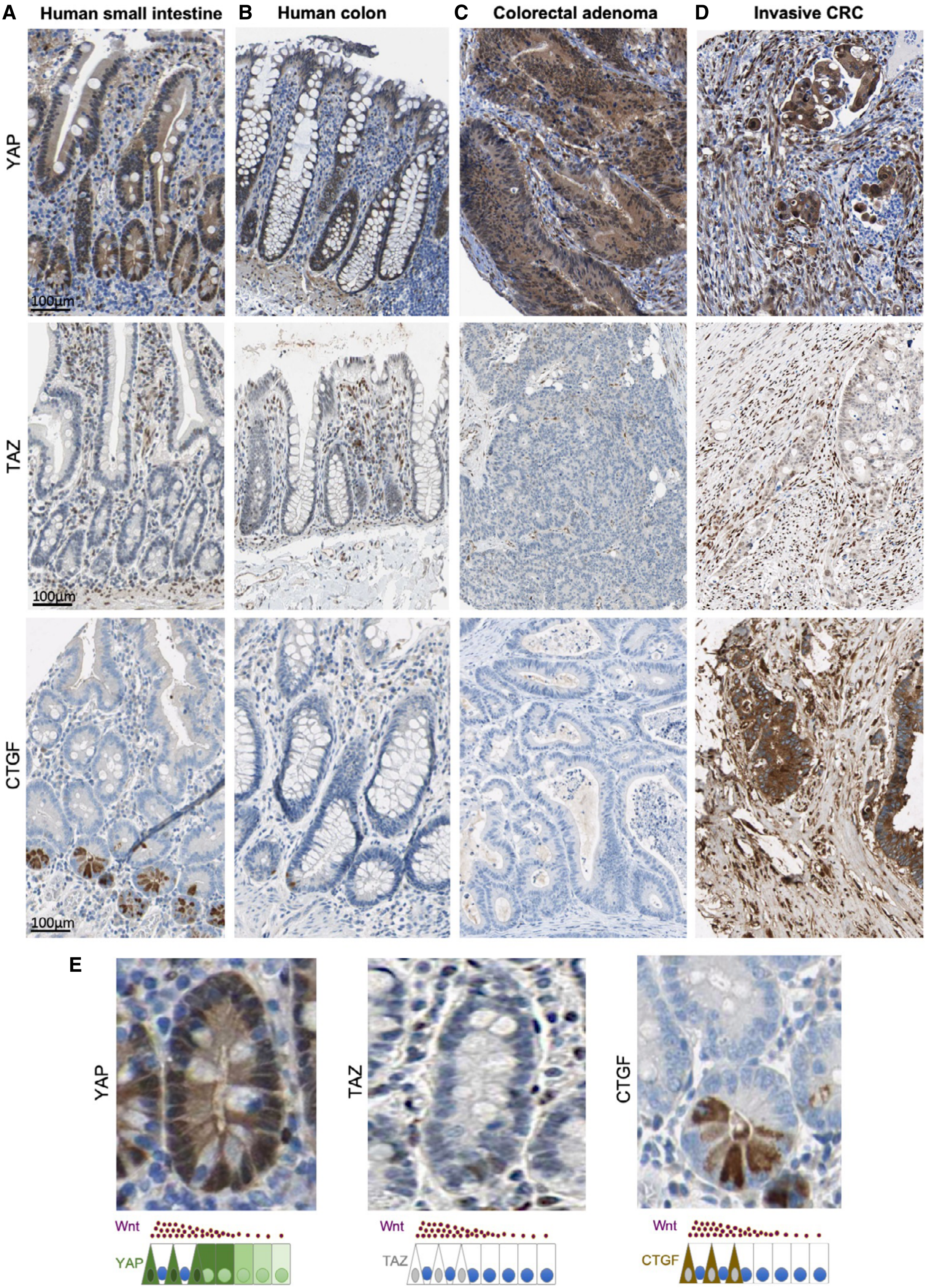
A Human small intestine
B Human colon
C Colorectal adenoma
D Invasive CRC
YAP
TAZ
CTGF
100µm
100µm
100µm
E
YAP
TAZ
CTGF
Wnt
YAP
Wnt
TAZ
Wnt
CTGF

Figure EV1.

Figure EV2. *Yap*/*Taz* double knockouts exhibit reduced proliferation and abnormal regeneration after gamma irradiation in the small intestine.

A Murine small intestines from (Cre negative) and *Villin-Cre*^{ERt} *Yap*$^{fl/fl}$ *Taz*$^{fl/fl}$ animals (*Yap*/*Taz* dKO) display a mildly increased rate of crypt cell apoptosis, marked by cleaved caspase 3 immunostaining, but no decrease in cell proliferation, marked by Ki67 immunostaining. Note that the images shown in this control are identical to those shown in Fig 1A.

B Murine small intestines isolated 3 days after treatment with 12 Gy irradiation (3 dpi) upon *Yap*/*Yap* dKO show both increased apoptosis, marked by cleaved caspase 3 immunostaining, and reduced cell proliferation, marked by Ki67 immunostaining. $n = 5$ animals at 3 dpi.

 © 2021 The Authors

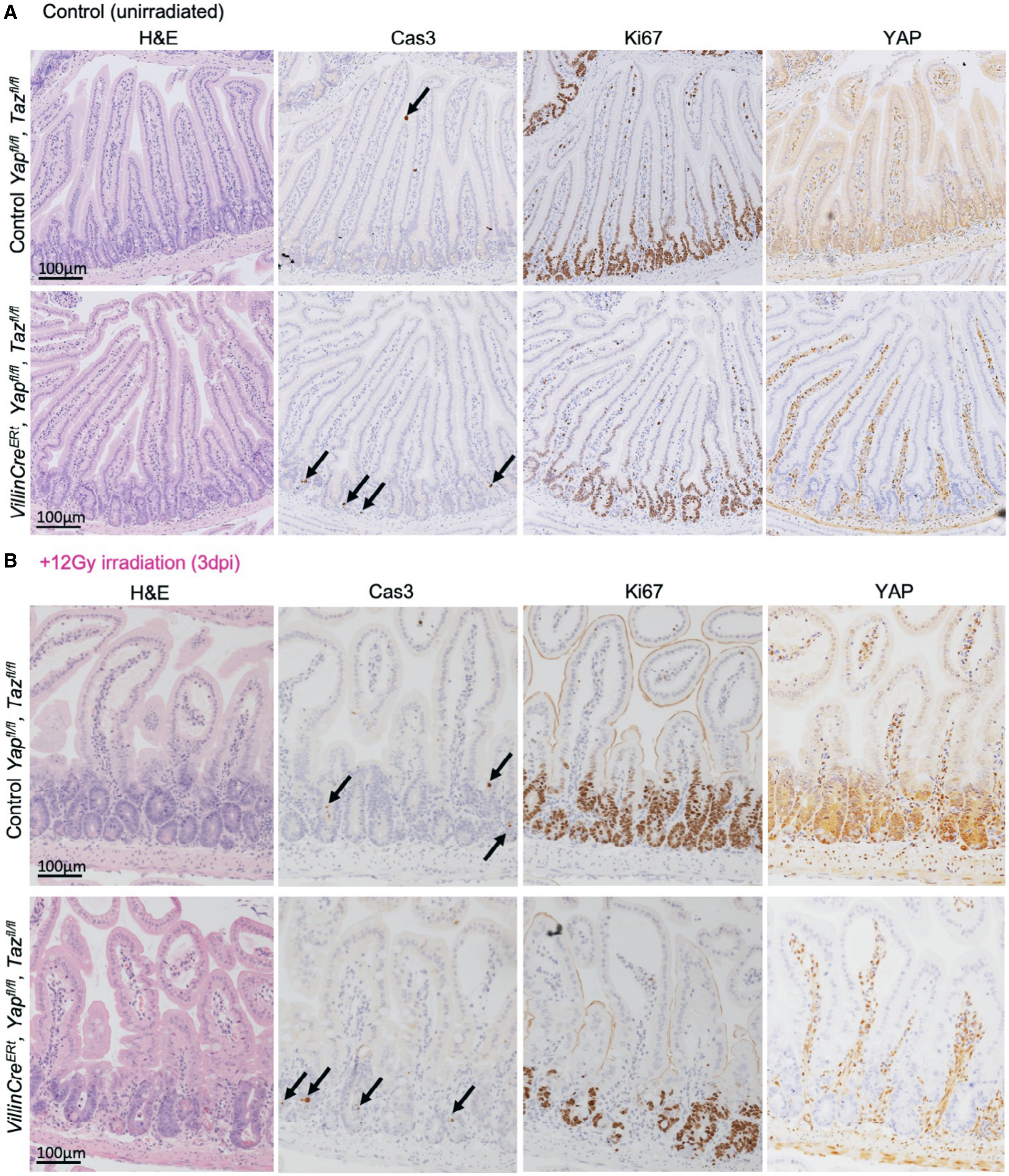
A
Control (unirradiated)
H&E
Cas3
Ki67
YAP
Control Yap^fl/fl, Taz^fl/fl
100µm
VillinCre^ERt, Yap^fl/fl, Taz^fl/fl
100µm
B
+12Gy irradiation (3dpi)
H&E
Cas3
Ki67
YAP
Control Yap^fl/fl, Taz^fl/fl
100µm
VillinCre^ERt, Yap^fl/fl, Taz^fl/fl
100µm

Figure EV2.

Figure EV3. ***Yap/Taz* double knockouts exhibit abnormal regeneration after gamma irradiation in the large intestine.**

A Murine colon from (Cre negative) and *Villin-Cre*^{ERt} *Yap*$^{fl/fl}$ *Taz*$^{fl/fl}$ animals (*Yap/Taz* dKO) display a mildly increased rate of crypt cell apoptosis, marked by cleaved caspase 3 immunostaining, but no decrease in cell proliferation, marked by Ki67 immunostaining. These figure panels are identical to those shown in Fig 1B and are shown for comparison only.

B Murine colon isolated 3 days after treatment with 12 Gy irradiation (3 dpi) upon *Yap/Taz* dKO show both increased apoptosis, marked by cleaved caspase 3 immunostaining, and reduced cell proliferation, marked by Ki67 immunostaining. n = 5 animals at 3 dpi.

© 2021 The Authors

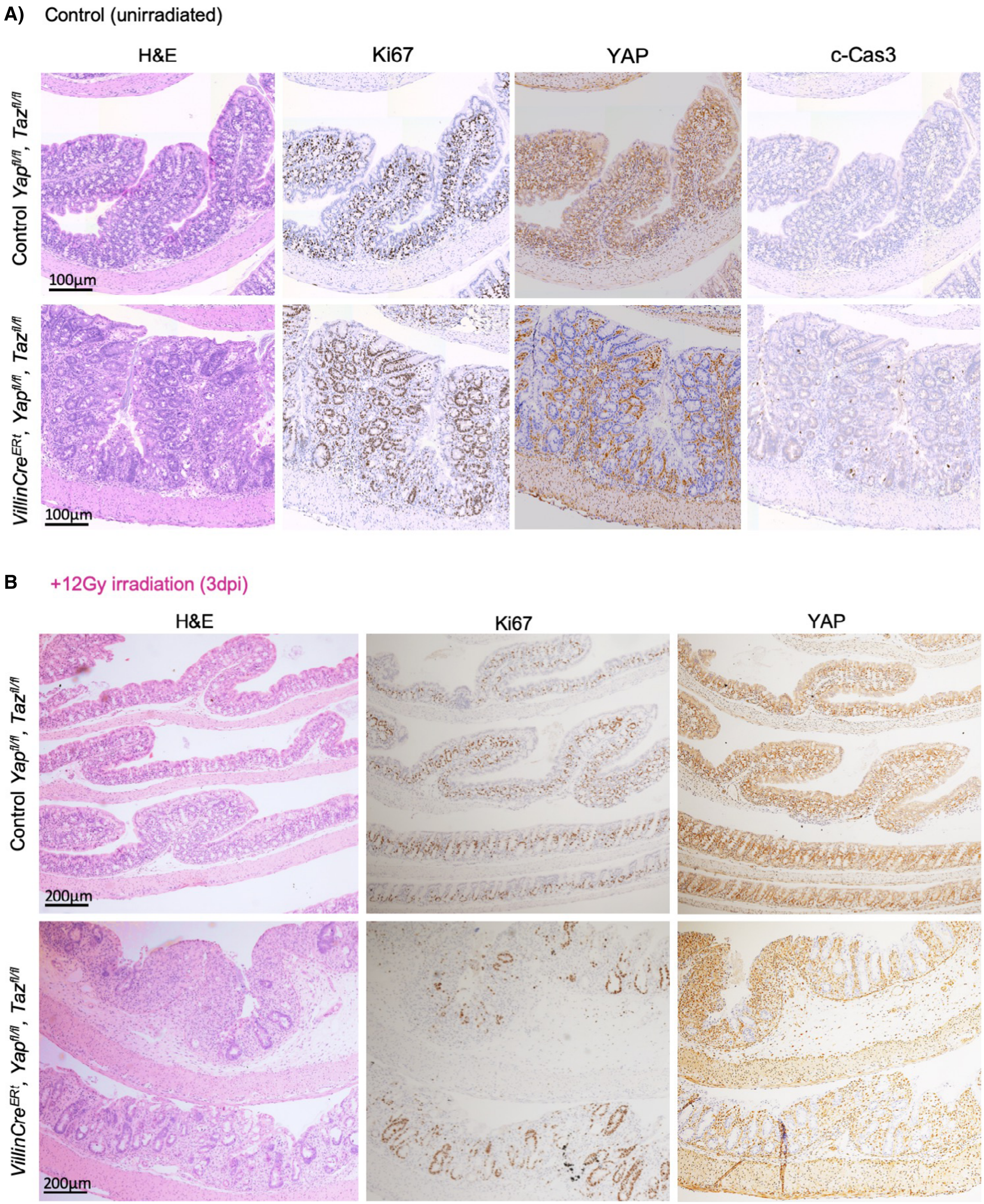
A) Control (unirradiated)
H&E
Ki67
YAP
c-Cas3
Control Yap^fl/fl, Taz^fl/fl
VillinCre^ERt, Yap^fl/fl, Taz^fl/fl
100µm
100µm
B +12Gy irradiation (3dpi)
H&E
Ki67
YAP
Control Yap^fl/fl, Taz^fl/fl
VillinCre^ERt, Yap^fl/fl, Taz^fl/fl
200µm
200µm

Figure EV3.

Figure EV4. *Lats1/2* double knockouts exhibit increased proliferation in both the small and large intestine, which can become ulcerated.

A Murine small intestines isolated from control (*Villin-Cre^{ERt}*) animals and *Villin-Cre^{ERt} Lats1$^{fl/fl}$ Lats2$^{fl/fl}$* double knockout *(Lats1/2 dKO)* animals immunostained for YAP and proliferation marker Ki67.

B Murine colons isolated from control (*Villin-Cre^{ERt}*) animals and *Villin-Cre^{ERt} Lats1$^{fl/fl}$ Lats2$^{fl/fl}$* double knockout *(Lats1/2 dKO)* animals immunostained for YAP and proliferation marker Ki67. Note region of colonic ulceration (red dotted line) arising at 7 days after 3× tamoxifen i.p. treatment. Inset shows quantification of the number of Ki67-positive cells per crypt in control and dKO animals. n = 5 animals for each genotypes.

 © 2021 The Authors

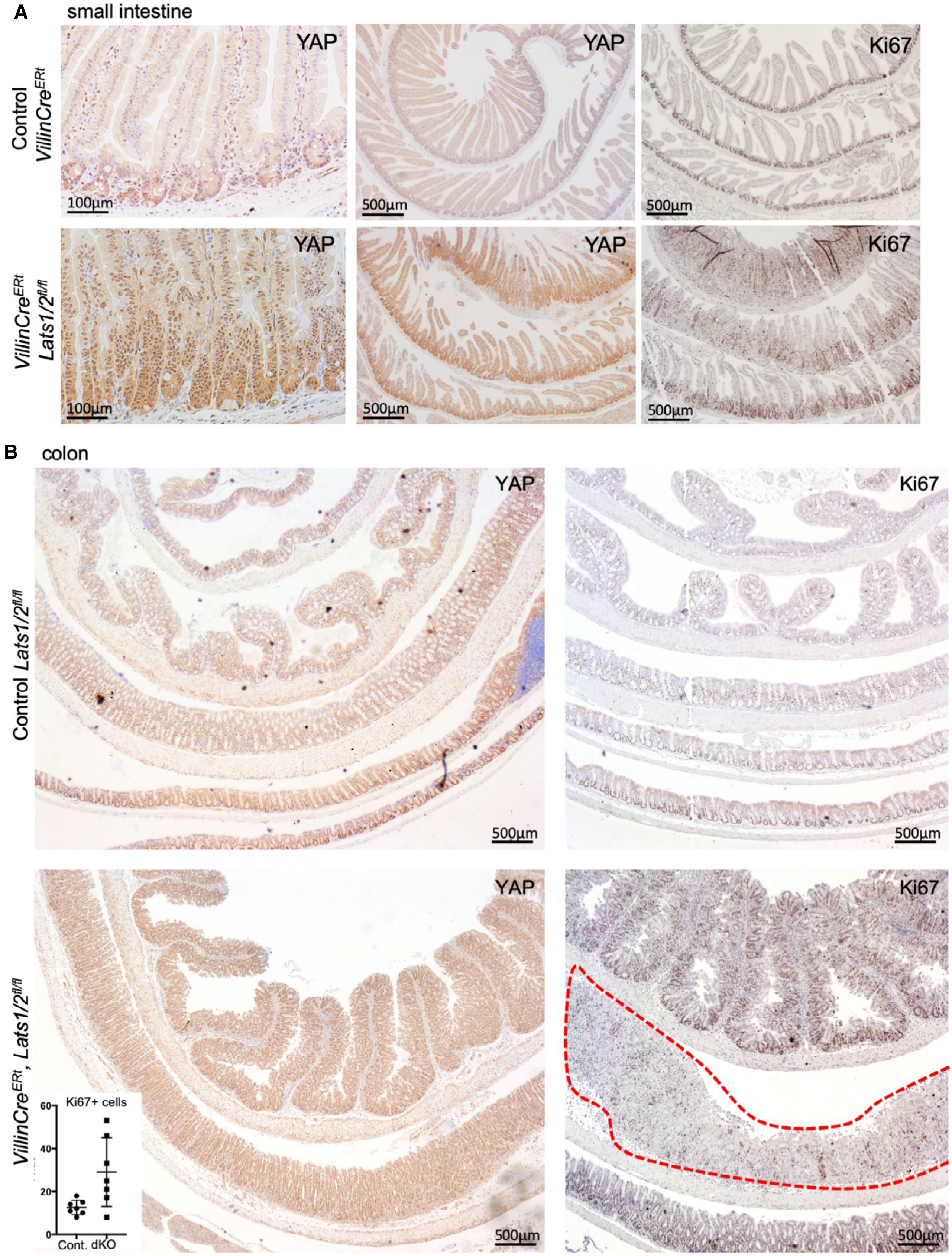
A
small intestine
Control VillinCre^{ERt}
VillinCre^{ERt} Lats1/2$^{fl/fl}$
YAP
YAP
Ki67
100µm
500µm
500µm
B
colon
Control Lats1/2$^{fl/fl}$
VillinCre^{ERt}, Lats1/2$^{fl/fl}$
YAP
Ki67
500µm
Ki67+ cells
60
40
20
0
Cont.
dKO

Figure EV4.

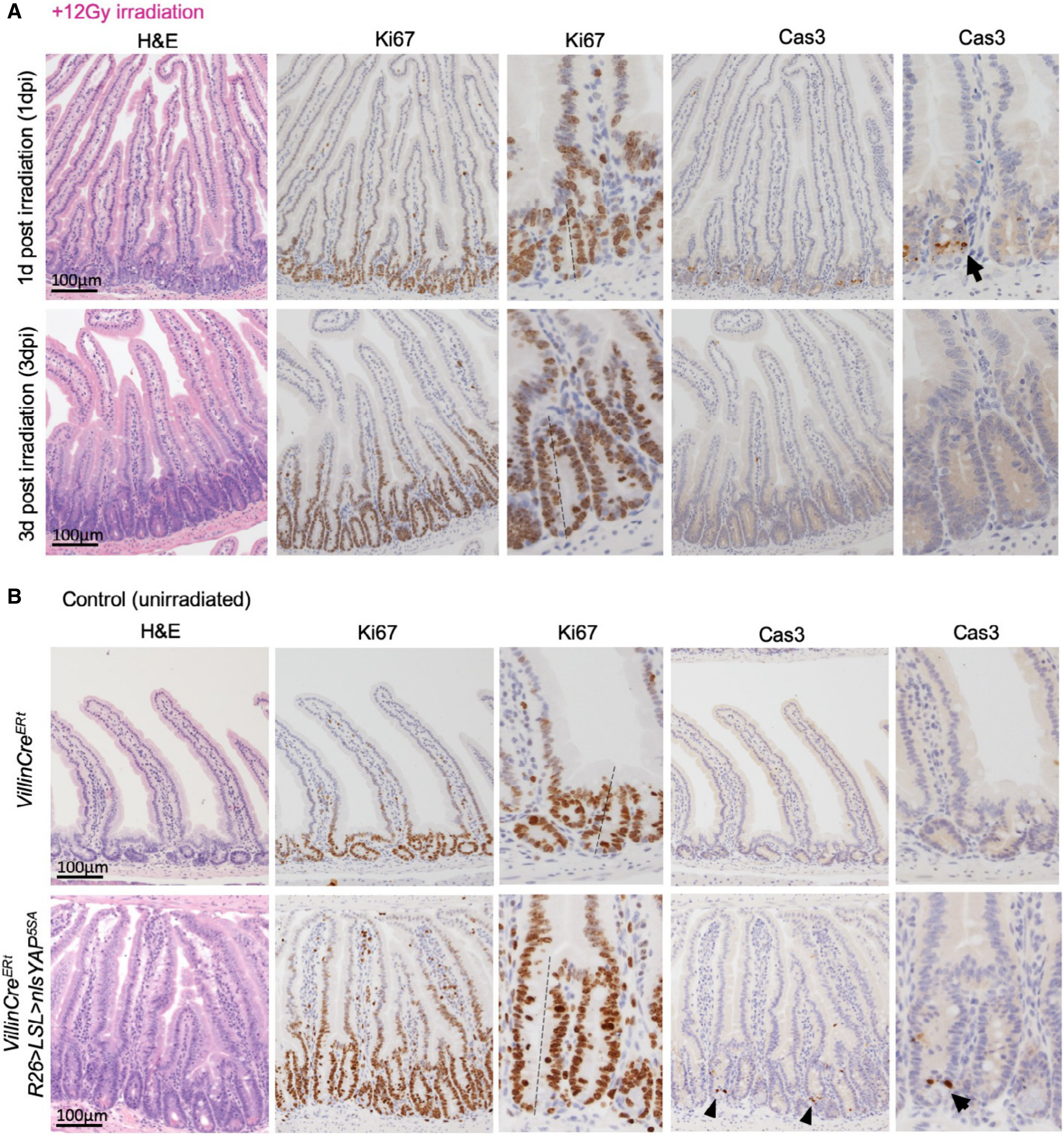

Figure EV5. Irradiation induces apoptosis followed by regenerative crypt hyperproliferation, a phenotype mimicked by ectopic expression of nuclear YAP.

A Murine small intestines treated with 12 Gy irradiation and isolated 1 day post-irradiation (dpi) and 3 dpi. Immunostaining for proliferation marker Ki67 and apoptosis marker cleaved caspase 3 (Cas3) is shown. n = 4 animals for each time point.

B Murine small intestines from control (*Villin-Cre*^{ERt}) and *Villin-Cre*^{ERt} *Rosa26*>*loxSTOPlox*>*nlsYAP*^{5SA} transgenic animals treated with 3× tamoxifen i.p. show a phenotype identical to that induced by irradiation in (A) after 5 days. n = 4 animals for each genotype.

 © 2021 The Authors
