# Supplementary figures and images for: Wnt and Src signals converge on YAP‐TEAD to drive intestinal regeneration
Source: EMBO J. 2021 May 5;40(13):e105770. doi: 10.15252/embj.2020105770 (PMC8246259; doi:10.15252/embj.2020105770)

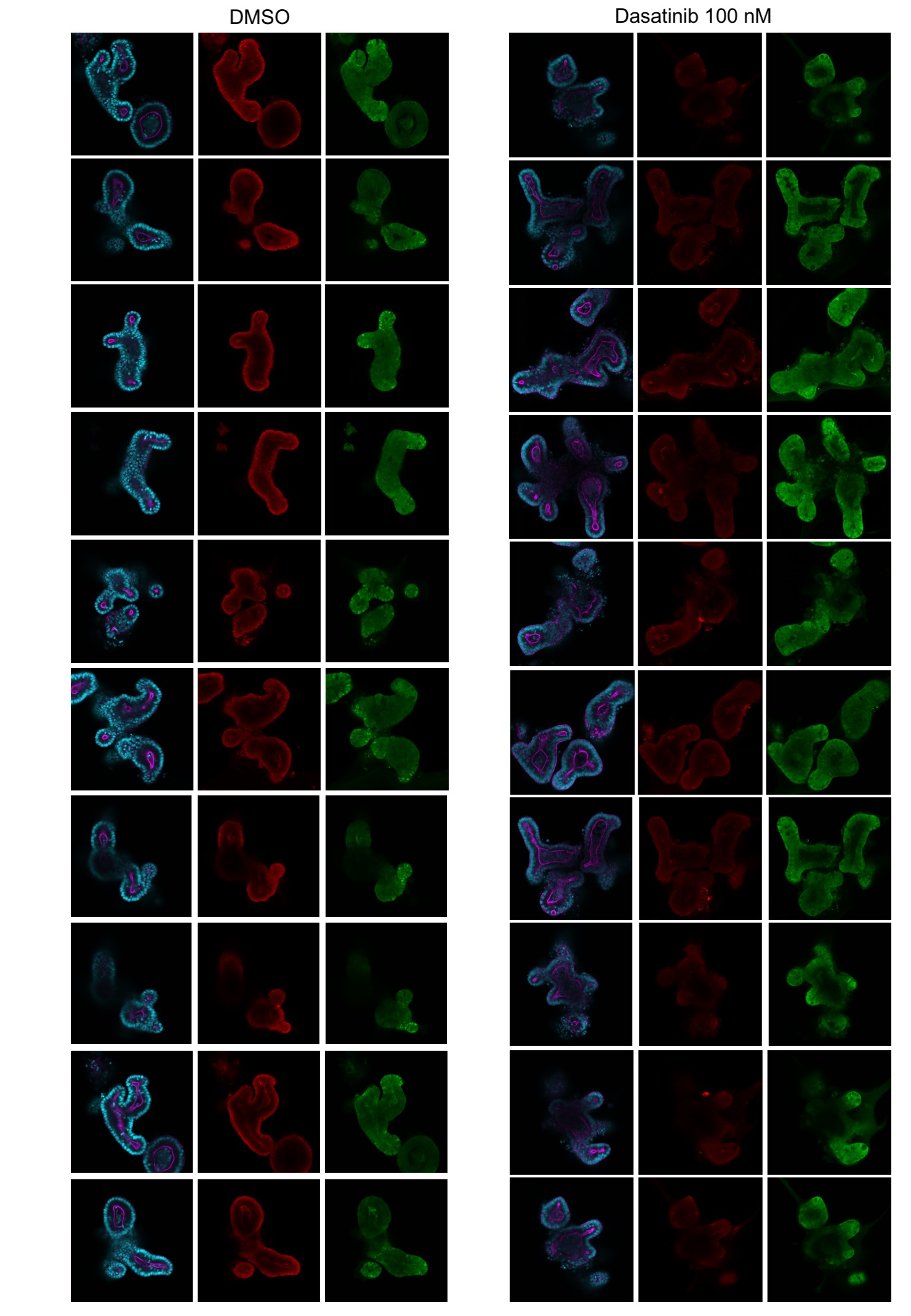

Supplement: Supplementary file 4 — Source Data for Figure 6 [file EMBJ-40-e105770-s005.jpeg]

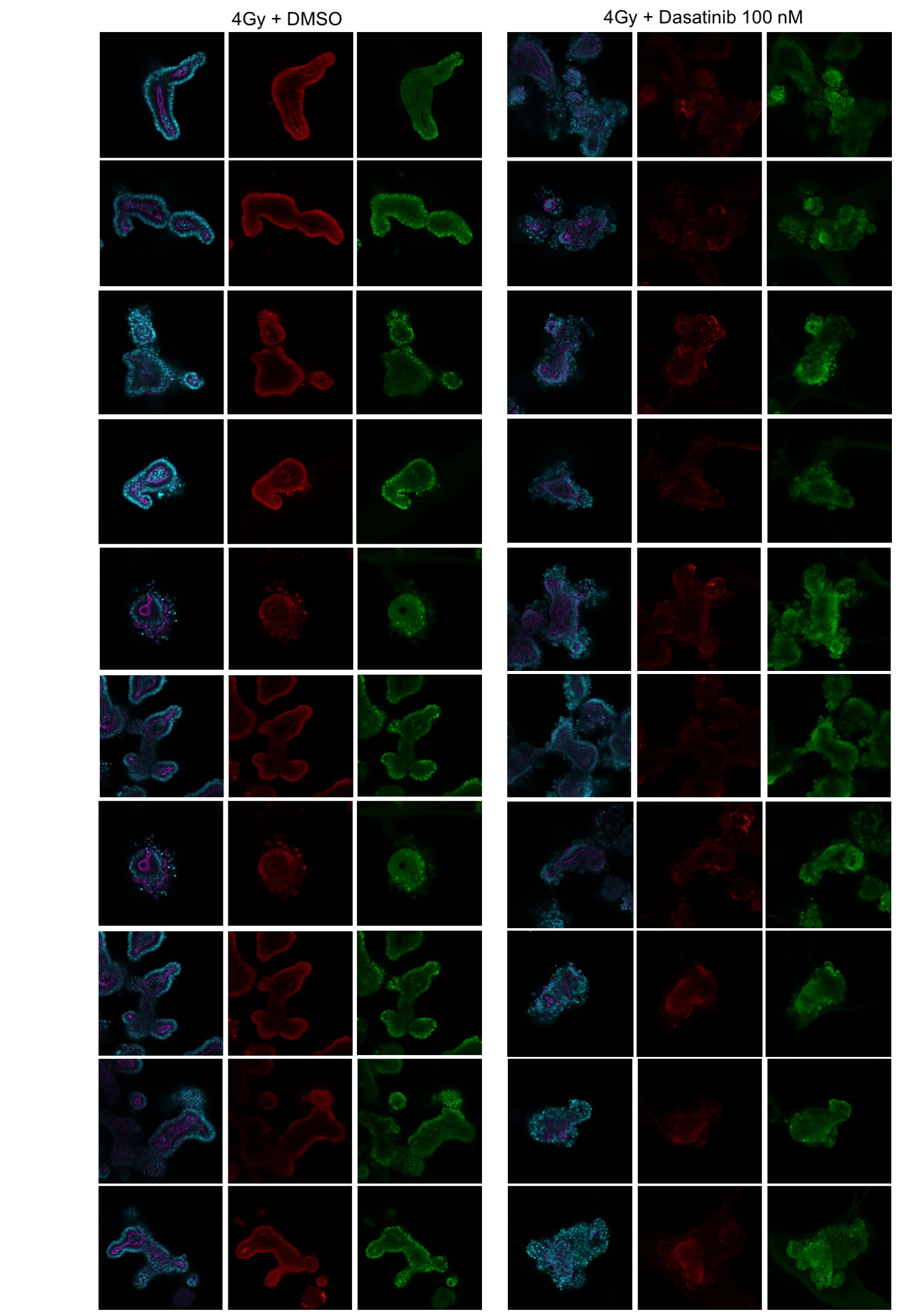

Supplement: Supplementary file 5 — Source Data for Figure 7 [file EMBJ-40-e105770-s004.jpeg]
